# Oxidative removal of stabilized landfill leachate by Fenton's process: process modeling, optimization & analysis of degraded products

**DOI:** 10.1039/c9ra09415f

**Published:** 2020-01-23

**Authors:** N. Jegan Durai, G. V. T. Gopalakrishna, V. C. Padmanaban, N. Selvaraju

**Affiliations:** Department of Civil Engineering, Kamaraj College of Engineering & Technology Madurai Tamilnadu India; Department of Civil Engineering, PSNA College of Engineering & Technology Dindugul Tamilnadu India gvtgkrishna@gmail.com +91 9943838272; Centre for Research, Department of Biotechnology, Kamaraj College of Engineering & Technology Madurai Tamilnadu India; Department of Biosciences and Bioengineering, Indian Institute of Technology Guwahati Assam India selva@iitg.ac.in +91 9446021424

## Abstract

In this study, the stabilized landfill leachate which has a BOD : COD ratio of 0.045 was treated using Fenton's process. The effect of process parameters like reaction time, pH, dose of FeSO_4_ and dose of H_2_O_2_ was estimated using One Factor At a Time (OFAT) and the linear, interactive and quadratic effects between the factors were studied using Face Centered Central Composite Design (CCF). In the OFAT approach, reaction time: 5 minutes, pH: 3.0, dose of FeSO_4_: 30 mM, and dose of H_2_O_2_: 30 mM were optimized. In CCF, the statistically optimized model shows maximum removal of organic substances at an FeSO_4_ concentration of 14.44 mM, pH 3.0 and 29.12 mM of H_2_O_2_. The regression co-efficient *R*^2^ = 0.9079, adj *R*^2^ = 0.854 and adequate precision = 14.676. The degradation of organic substances was assessed by measuring the Chemical Oxygen Demand (COD). Total Organic Carbon (TOC) and Gas Chromatography-Mass Spectroscopy (GC-MS) were investigated for the sample corresponding to the maximum COD reduction.

## Abstract 2

In this study, the stabilized landfill leachate which has a BOD : COD ratio of 0.045 was treated using Fenton's process.

## Graphical abstract

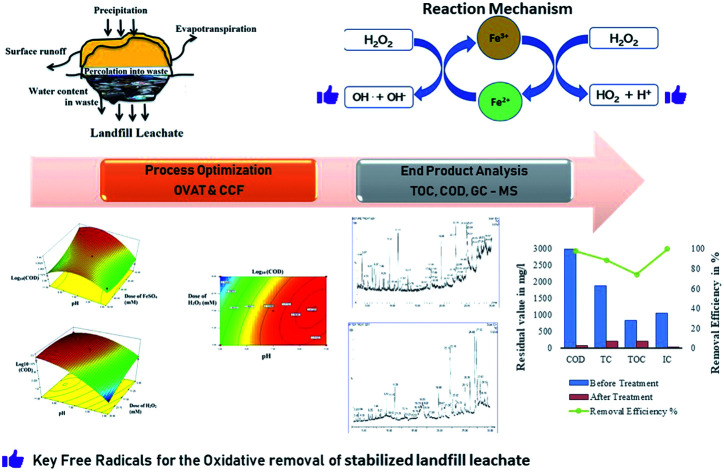

## 1. Introduction

Leachate is a liquid extract which oozes out of solid waste, due to degradation of the solid waste and due to percolation of rain water through the waste.^1–3^ Due to percolation of leachate through the soil and migration of leachate by surface runoff contaminates the soil, groundwater and surface water bodies in and around the landfill site.^4,5^ The leachate contains various organic substances (both biodegradable and non-biodegradable), inorganic substances (chloride, magnesium, sodium, potassium, ammonia nitrogen, bicarbonates *etc.*), heavy metals, and toxic substances.^6,7^ The volume of leachate production and the concentration of the contaminants present in the leachate vary due to the composition of the constituents present in the solid waste, method of landfill, age of solid waste landfill, seasonal changes (change in temperature, rainfall pattern), and environmental conditions of the landfill (aerobic, anaerobic & facultative).^6,8^ The characteristics of the leachate determine a suitable method of treatment that can be adapted to the specific leachate.^3^ Young leachate (age of landfill 1–2 years) where the biochemical oxygen demand (BOD_5_)/chemical oxygen demand (COD) is >0.6 is amenable for biological treatment. Stabilized leachate (age of landfill 5–10 years) and a BOD_5_/COD ratio < 0.3 can be treated by physicochemical treatment methods.^9,10^

Leachate contains trace organic contaminants (OC), which have high impact on environment and living beings and it cannot be removed completely by conventional treatment (biological treatment and physical/chemical treatment) or membrane treatment, while the membrane bioreactor is capable of removing these contaminants completely but it is expensive.^11^ Advanced Oxidation Process (AOP) was capable of destructing bio refractory compounds in wastewater.^10^ Common AOP was carried out by different combinations of oxidants (O_3_, H_2_O_2_, and combination of both), catalyst (metal salt/electrodes) and irradiation methods (ultraviolet radiation, visible light, ultrasonic sound and microwave).^12–14^ In the Fenton process the catalyst ferrous ion combines with oxidizing agent hydrogen peroxide under acidic condition and produces OH radicals which oxidize the organic substance present in the wastewater as the reaction shown in eqn (1).^5,15^ The hydroxide radicals have higher oxidation potential ((*E*_0_ = 2.80 V) than ozone (*E*_0_ = 2.07 V)).^16^ Ferric ion generated in this process combines with hydrogen peroxide and regenerates the catalyst ferrous ion as the reaction shown in eqn (2).^5,15^

1 Fe^2+^ + H_2_O_2_ →Fe^3+^ + ·OH + OH^−^

2 Fe^3+^ + H_2_O_2_ →Fe^2+^ + ·HO_2_ + H^+^

Among various AOP Fenton process was found to be a simple, cost effective and eco-friendly method since it doesn't require electric energy/UV lamp/ultrasonic device.^4,17^ Fenton process can be used as pretreatment method to biological process or post treatment to reduce the organic content to the desired level.^8^ The maximum COD removal of 80% was achieved for leachate at Fe^2+^/H_2_O_2_ molar ratio of 1/13.3 within 5 minutes.^2^ 82% of phenol was degraded by Fenton's process under pH 3, Fe^2+^/H_2_O_2_ molar ratio of 0.026 after 45 minutes.^18^ The efficiency of the process depends on the operating parameters such as reaction time, initial pH of the waste water, dosage of catalyst and dosage of oxidant.^19^ The correct combination of these parameters is required to attain the high treatment efficiency. The optimum condition of different operating parameters on oxidation and mineralization of various substances were greatly influenced by the nature (homogeneous/heterogeneous) and complexity (bond between the components) of the organic substance.^8^ Usually the optimization was carried out by keeping all other parameters as constant and varying only one variable. This method of optimization consumes more time and energy. But the results obtained by this approach may not be optimum because the interaction among the parameters was ignored.^20^ The above issue can be solved by optimizing the parameter by Response Surface Methodology (RSM).

While inferring the various research articles, very less work have reported about the trace organic substance, most of the researchers reported the efficiency of the treatment process in terms of reduction in COD, color and TOC. Moreover the trace organic substance has a significant impact on living beings, environment and affect the biological treatment process as these substance was complex, toxic and non-biodegradable. Fenton's process was a technology successfully demonstrated to treat wide range of pollutants present in municipal and various industrial effluents.^21^ The objective of this research work is to investigate the removal of organic substance from leachate by Fenton process. The effect of the different operating parameters on removal of organic substance was carried out by one variable approach. The interaction among the operating parameters was optimized by response surface methodology. The reduction of organic substance after treatment of leachate was examined by measuring the residual COD. The Gas Chromatography Mass Spectrum (GC-MS) analysis and TOC analysis was carried out for the leachate sample before treatment and after treatment for the sample corresponding to maximum reduction of residual COD under optimized condition of Fenton's process to evaluate the amount of reduction in trace organic substance, total carbon, organic carbon, inorganic carbon.

## 2. Materials and methods

### 2.1 Leachate collection

Leachate samples were collected from Madurai corporation compost and landfill yard, Avaniyapuram Municipality, Madurai, Tamilnadu, India. It receives around 600 to 750 metric tons of solid waste every day. Samples were collected in polyethylene containers and stored in freezer at 4 °C. The characterization and treatment studies were carried out at room temperature.

### 2.2 Reagents

The reagents used for this study are phenolphthalein, methyl orange, manganous sulphate, sodium iodide, sodium azide, starch, sodium thio-sulphate, ferrous sulphate, hydrogen peroxide (30% v/v), sodium hydroxide, concentrated hydrochloric acid, silver sulphate, mercuric sulphate, potassium dichromate, concentrated sulphuric acid, ammonium ferrous sulphate (Merck, India).

### 2.3 Analytical method

The characteristics of leachate was analysed as per standard methods.^22^ The residual COD of all samples after Fenton process was performed by closed reflux method with titrimetric (APHA, Method 5220 C). Total solids were measured by drying the sample at 103–105 °C (APHA, Method 2540 B). Fixed and volatile solids were determined by igniting the sample at 550 °C (APHA, Method 2540 E). Total suspended solids was measured by filtering and drying the residue deposited in the glass fibre filter paper at 103–105 °C (APHA, Method 2540 D), (APHA, Method 2540 B). Turbidity was determined by nephelometric method (APHA, Method 2130 B). The colour was visually determined. APHA, Method 5210 B was performed to determine BOD_5_. Alkalinity of the sample was evaluated by titration method (APHA, Method 2320B). TOC was analysed by combustion-infrared method (APHA, Method 5310 B) using TOC analyzer (Analytikjena/multi N/C 3100). In this study, the DOC is assumed to be equivalent to TOC since the sample was processed at 80 °C. GC-MS was carried out to identify the trace organic contaminants using the instrument PerkinElmer Clarus SQ8C. 30 mL sample was mixed with equal volume of diethyl ether and the layer of diethylether was separated and dried. 2 mL of ethanol was added to the dried sample, subjected to analysis. The equipment consists of standard capillary non-polar column (30 M length, 0.25 mm id). The flow rate in the GC was 1 mL min^−1^ with a carrier gas (helium).

### 2.4 Degradation of landfill leachate by Fenton's process – OFAT

Fenton experiment was carried out by batch process at room temperature under atmospheric pressure. Initially the reaction time was optimized by adjusting the leachate samples to pH 3.0 and 100 mL of sample was taken in a beaker; 20 mM hydrogen peroxide (H_2_O_2_) and 20 mM ferrous sulphate (FeSO_4_) granules were added and stirred. At different time interval (1, 3, 5, 10, and 15 minutes) the sample was drawn and sodium hydroxide solution was added immediately to increase the pH of the sample to 8, which stops the oxidation process, the residual H_2_O_2_ was determined by potassium permanganate titrimetric method and the unreacted residual H_2_O_2_ was removed by heating the sample at 50 °C for 30 minutes,^9^ then the sample was centrifuged at 10 000 rpm and residual COD of the centrifuged sample was measured. For the optimized reaction time, the effect of initial pH was studied at different pH ranging from 3.0 to 11.0. The pH of the sample was modified using 1 N hydrochloric acid and 1 N sodium hydroxide. The effect of dose of FeSO_4_ and H_2_O_2_ was studied at variable range of FeSO_4_ dosages (10 mM to 50 mM) and hydrogen peroxide dosages (10 mM to 30 mM).

### 2.5 Degradation of landfill leachate – experimental design and statistical model

The optimization of the process variables and the linear, interactive and quadratic effects on the process was studied through CCF.^23,24^ In this study three independent variables *A*: pH, *B*: hydrogen peroxide (H_2_O_2_) dose, *C*: ferrous sulphate (FeSO_4_) dose and residual COD as response was evaluated by 20 experiments (2^*n*^ + 2*n* + *x*_0,_ 2^3^ = 8 – factorial points; 2 × 3 = 6 – axial points; 6 – centre points). The Table 1 shows the summary of design parameters for degradation of landfill leachate by oxidative Fenton's process with respect to actual and coded factors for CCF design. Design Expert 7.0.0., Stat-ease, USA was used to design the experiments and to analyze the data.

Experimental design for the degradation of landfill leachate by oxidative Fenton's process

**Table:** 

Factor	Unit	Low actual	High actual	Mean	Std Dev.
*A* = pH	—	3.00	11.00	7	2.828
*B* = dose of H_2_O_2_	mM	5.00	30.00	17.5	8.839
*C* = dose of FeSO_4_	mM	10.00	50.00	30.0	14.142

**Table:** 

Response	Unit	Model	Transformation	Mean	Ratio
*Y*_1_ = COD	mg L^−1^	Quadratic	Base 10 log	812.04	36.2670

## 3. Results and discussion

### 3.1 Characteristics of leachate

The characteristics of the landfill leachate were shown in Table 2. While comparing the characteristics with the literatures, it is evident that the leachate is stabilized leachate.^4,10^ The pH of leachate is alkaline and the BOD_5_ to COD ratio is 0.045 which indicates that the physico-chemical treatment is suitable for the treatment of leachate.^5^

**Table:** Characteristics of leachate

Parameters	Values
pH	8.8
Alkalinity	2000 mg L^−1^
Total solids	19 000 mg L^−1^
Total volatile solids	8500 mg L^−1^
Total fixed solids	10 500 mg L^−1^
Total suspended solids	1600 mg L^−1^
Turbidity	400 NTU
Colour	Dark brown
TOC	826 mg L^−1^
COD	2990 mg L^−1^
BOD_(5,20)_	135 mg L^−1^
BOD_(5,20)_/COD	0.045

### 3.2 Treatment of landfill leachate by Fenton process

#### 3.2.1 Effect of reaction time

**Fig. 1:**
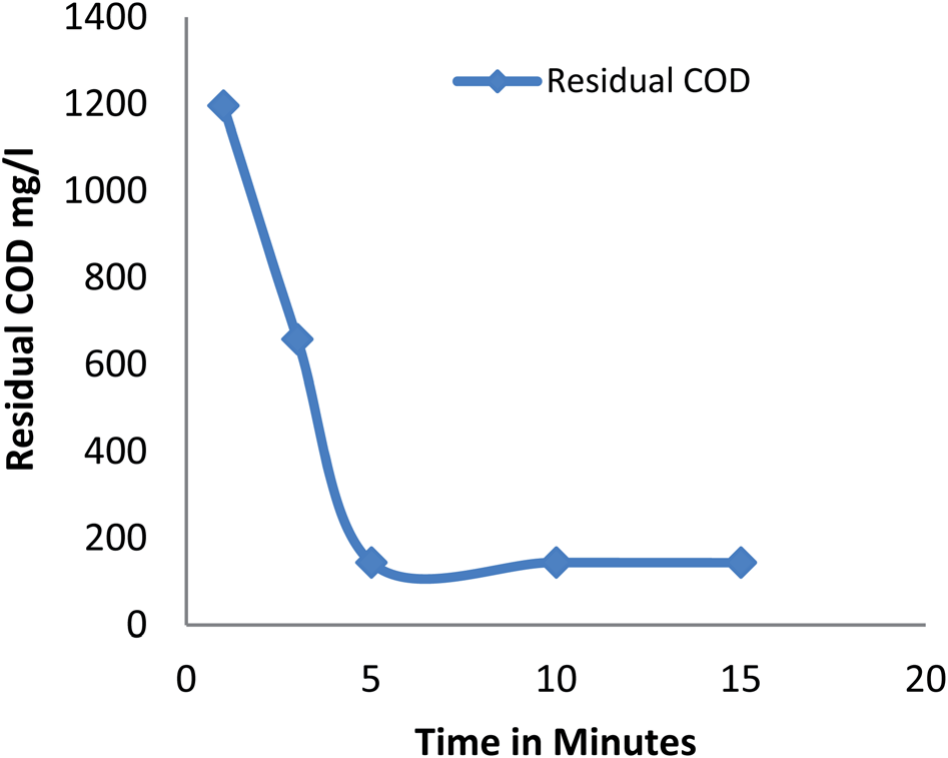
Residual COD after Fenton process: effect of reaction time (pH: 3; H_2_O_2_ dosage: 20 mM; Fe^2+^ dosage: 20 mM).

Fig. 1 shows the amount of residual COD after the process at different reaction time Fenton process for a constant dose of hydrogen peroxide (20 mM), Fe^2+^ (20 mM) at pH 3. Residual COD decreases as the time increases from 1 to 5 minutes and remains constant after 5 minutes. It was observed that 60% of COD removal was observed in first minute; the initial rapid oxidation was due to generation of hydroxide radicals by the ferrous ion catalyst^25^ and only additional COD removal of 19% to 37% was observed at a time period of 3 minutes and 5 minutes respectively, gradual decrease of oxidation was due to less availability of ferrous ion catalyst to generate hydroxide radical.^25^ Maximum COD removal was achieved in 5 minute. The results were in accordance with several literatures that is the degradation efficiency is very rapid within 15 minutes of the reaction.^26^

#### 3.2.2 Effect of pH on residual COD

**Fig. 2:**
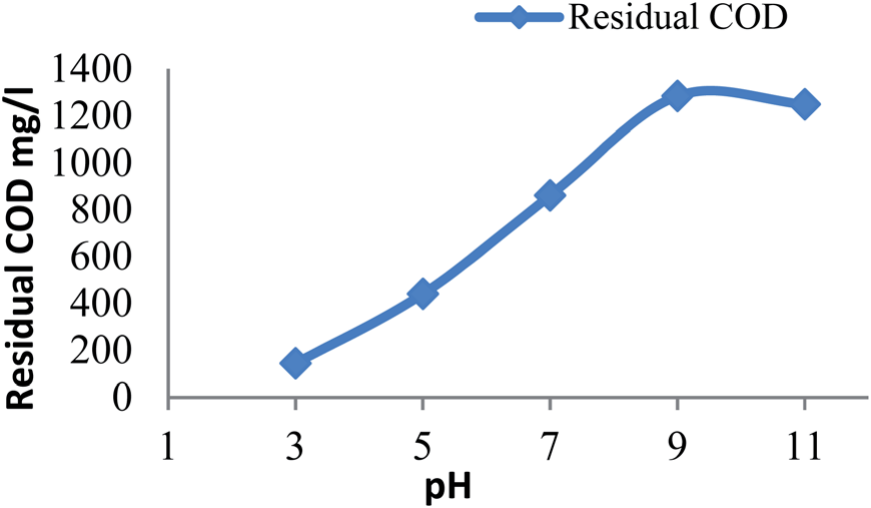
Residual COD after Fenton process: influence of pH (H_2_O_2_ dosage: 20 mM; Fe^2+^ dosage: 20 mM).

Fig. 2 shows the amount of residual COD remains after Fenton process at different initial pH of leachate for a constant dose of hydrogen peroxide (20 mM) and Fe^2+^ (20 mM). The residual COD increases from acidic to alkaline pH. At higher pH inactive iron oxohydroxides and ferric hydroxide precipitate was identified.^13^ The ferric hydroxide precipitate catalyzes the decomposition of H_2_O_2_ to O_2_ and H_2_O decreases the formation of hydroxide radicals^27^ reduces the oxidation process. The efficient pH was found to be pH 3. In acidic range the oxidation leading the Fenton process but at alkali range the coagulation predominant the Fenton process it was due to the formation of ferric hydroxide precipitate.^9^

#### 3.2.3 Effect of hydrogen peroxide on residual COD

**Fig. 3:**
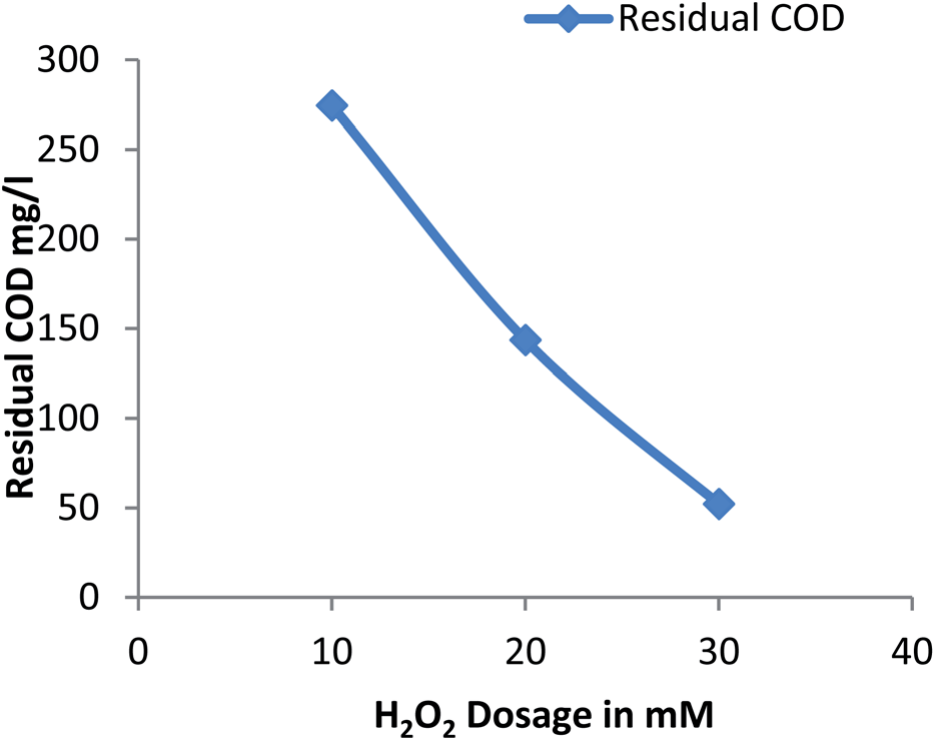
Residual COD after Fenton process: influence of H_2_O_2_ dosage (pH: 3, Fe^2+^ dosage: 20 mM).

Fig. 3 shows the residual COD values for different dose of hydrogen peroxide at a constant pH: 3 and Fe^2+^ dosage: 20 mM. The residual COD decreases with increase in hydrogen peroxide from 10 mM to 30 mM. The dosage of hydrogen peroxide was limited as the unused portion of hydrogen peroxide creates harmful effect on the organisms and scavenging of hydroxide radicals generated due to large amount of hydrogen peroxide.^13^

#### 3.2.4 Effect of ferrous ion on residual COD

**Fig. 4:**
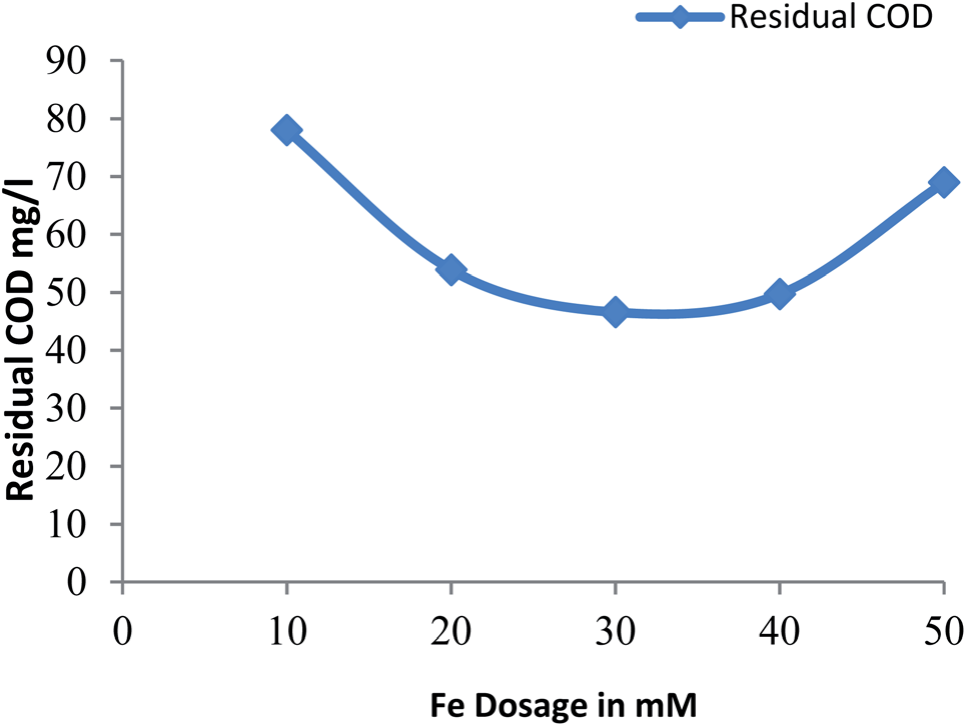
Residual COD after Fenton process: influence of Fe^2+^ dosage (pH = 3, H_2_O_2_ dosage = 30 mM).

Fig. 4 shows the residual COD values for different dose of Fe^2+^ at a constant pH: 3 and hydrogen peroxide dose: 30 mM. The residual COD decreases for Fe^2+^ dose of 10 mM to 30 mM and residual COD increases with further increase in dose of Fe^2+^, due to scavenging of generated hydroxide radicals due to excess amount of ferrous ion with further increase in dose of Fe^2+^.^28^ More reduction in residual COD was observed for Fe^2+^ dose of 30 mM.

### 3.3 Process optimisation by face centred central composite design

The mathematical relationship between the process variables and response with respect to concentration of residual COD is shown in the eqn (3). Table 3 shows the adequacy of the model and it was evaluated using the analysis of variance (ANOVA). The significance of the model terms were explained in Table 4.

**Table:** Analysis of variance (ANOVA) for the degradation of landfill leachate by oxidative Fenton's process^a^

Source	Sum of squares	Df	Mean square	*F* value	*p*-Value prob > *F*
Model	2.88	7	0.41	16.90	<0.0001
*A* – pH	1.22	1	1.22	50.03	<0.0001
*B* – dose of H_2_O_2_	0.48	1	0.48	19.88	0.0008
*C* – dose of FeSO_4_	0.13	1	0.13	5.28	0.0403
*AB*	0.37	1	0.37	15.29	0.0021
*AC*	0.19	1	0.19	7.91	0.0157
*A* ^2^	0.47	1	0.47	19.18	0.0009
*C* ^2^	0.091	1	0.091	3.75	0.0765
Residual	0.29	12	0.024		
Lack of fit	0.29	7	0.042	387.12	<0.0001
Pure error	0.00054	5	0.000107		
Cor total	3.17	19			

a *R* ^2^: 0.9079; adj *R*^2^: 0.8542; adeq precision: 14.676; CV%: 5.63.

**Table:** Significance of the model – parameter terms of RSM–CCF

Parameters	Terms & values	Significance with respect to process
Positive effects	Linear effect of *A*	Increase in the value of *A*, *AB*, *C*^2^ will improve the performance of the process
	Interactive effect of *AB*	
	Quadratic effect of *C*	
Negative effects	Linear effect of *B*	Increase in the value of *B*, *C*, *AC* and *A*^2^ will decrease the performance of the process
	Linear effect of *C*	
	Interactive effect of *AC*	
	Quadratic effect of *A*	
*P*-Value	<0.0001	Model is significant to explain the process
*F*-Value	16.90	
Regression coefficient	*R* ^2^ = 0.9079	The closeness of adjusted *R*^2^ with *R*^2^ explains the new parameters improves the model more than the expected probability
	Adjusted *R*^2^ = 0.8542	
Coefficient of variation	5.63%	The variability of data in sample with respect to the mean of the population is 5.63% and 94.37% of the model prediction is similar and replicable
Adequate precision	14.674	The predicted response by the mathematical equation is developed by the actual appropriate signals

Before transformation:

Equation based on real factors:

3 COD = +885.512 + 96.027 × *A* − 16.913 × *B* − 14.989 × *C*

**Fig. 5:**
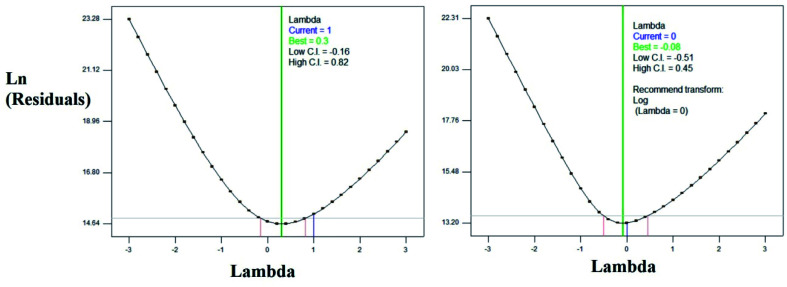
BOX–COX Plots (a) before transformation (b) after log_10_ transformation.

From the ratio of maximum and minimum experimental values, the log_10_ transformation was carried out as proposed by BOX–COX plot. The plots were shown in the Fig. 5. The transformed equation is given in the eqn (4).

After transformation:

4 log_10_(COD) = +2.07706 + 0.40426 × *A* − 0.047816 × *B* − 0.017455 × *C* + 0.00431592 × *A* × *B* − 0.00193959 × *A* × *C* −0.02388 × *A*^2^ + 0.00042265 × *C*^2^

**Fig. 6:**
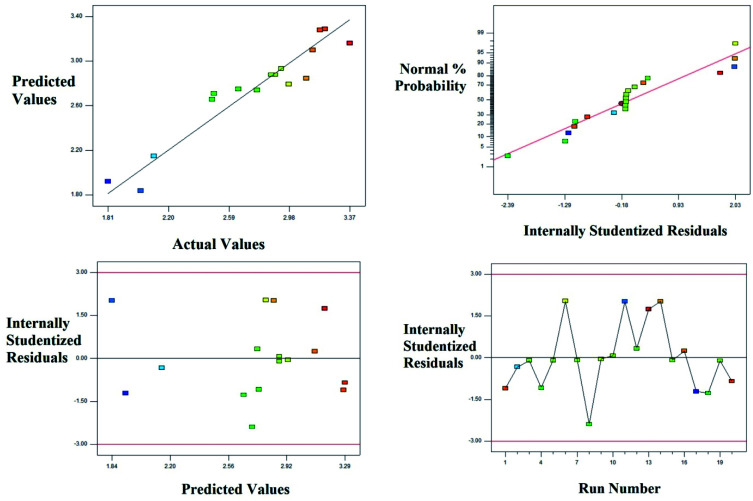
Degradation of the landfill leachate by oxidative Fenton's process; plot of the experimental *vs.* predicted values and its relative residual plots.

The diagnostic, normal probability and the residual plots which relates the experimental and predicted values are shown in the Fig. 6. The Table 4 and Fig. 6 explain the fitness of the model explaining the pattern of degradation of landfill leachate.

#### 3.3.1 Interactive effect between the process parameters

The interactive and quadratic effect between the processes variables could be studied using the equation developed using the CCF modeling. The interaction between the parameters were better studied using 2D contour plots or 3D surface plots.^29,30^

**Fig. 7:**
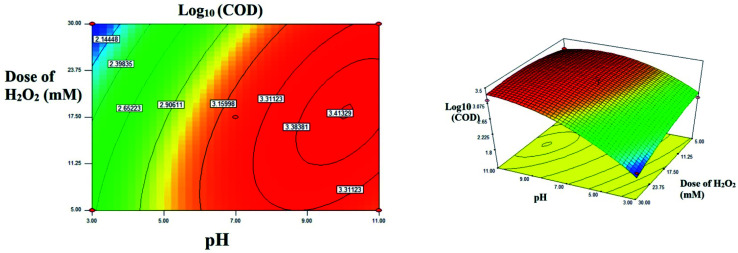
The contour plot and the 3D response surface plot of the degradation of landfill leachate by oxidative Fenton's process as the function of pH and dose of H_2_O_2_. Dose of FeSO_4_ = 10 mM.

The interactive effect of *AB*: pH *vs.* H_2_O_2_ dose: the 2D and 3D plots were shown in the Fig. 7. At lower dose of H_2_O_2_ in the range of 5 mM, as the pH increases from acidic range (3.0) to alkaline range (11.0), approximately 3.7 fold increases in residual COD was observed. Interestingly, at higher doses of H_2_O_2_ (30 mM), nearly 28 fold increase was observed in residual COD as the pH increases towards alkaline. It is inferred that at lower dose of H_2_O_2_ pH doesn't show much significant difference, whereas at higher dose of H_2_O_2_, acidic pH will enhance the rate of reaction in several folds. This observation is also evident from the interactive effect of pH & dose of H_2_O_2_. At acidic pH: 3.0, residual COD decreases to 68.86 mg L^−1^ (log_10_ = 1.858) from 512.80 mg L^−1^ (log_10_ = 2.710). No much significant difference was observed at alkaline pH. In acidic range of pH 2.0 to 4.0, higher OH radicals were generated or the reaction rate increased by a reaction involving in the organometallic complex.^10^ At higher pH (>5) acceleration of auto decomposition of hydrogen peroxide takes place and oxidation potential of OH radicals decreases. The excess amount of hydrogen peroxide added reduces the oxidation of organic substance due to scavenging of hydroxide radicals by unused portion of hydrogen peroxide in the Fenton process.^13^

**Fig. 8:**
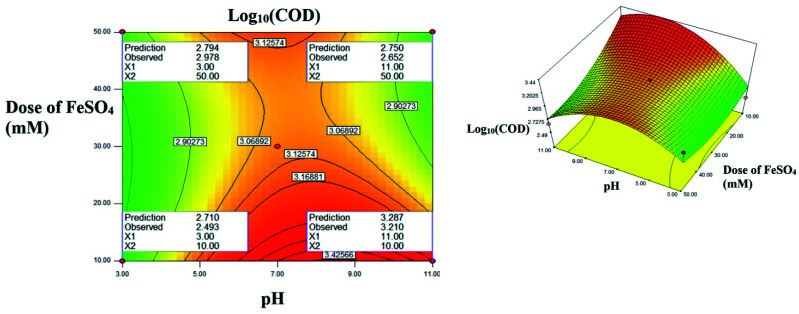
The contour plot and the 3D response surface plot of the degradation of landfill leachate by oxidative Fenton's process as the function of pH and dose of FeSO_4_. Dose of H_2_O_2_ = 10 mM.

The interactive effect of *AC*: pH *vs.* FeSO_4_ dose: Fig. 8 explains the interactive effect. The residual COD increases when the initial concentration of FeSO_4_ and increase of pH ranging from 3.0 to 11.0. At acidic pH, no significant change in residual COD was observed. Interestingly at alkaline pH 3.4 fold decreases was observed as concentration of FeSO_4_ increased. The maximum residual COD reduction was observed in the lower initial FeSO_4_ concentration. At higher pH range the formation of relatively inactive iron oxo hydroxides and ferric hydroxide precipitate, the activity of Fenton reagent is reduced due to lesser hydroxide radicals are generated and due to less availability of free iron ions.^13^

#### 3.3.2 Optimization of the process variables and confirmation of reduction in residual COD concentration

From the results of OFAT and the interactive effect studies through CCF, the process factors for the degradation of leachate were optimized using numerical optimization method. To achieve maximum reduction in the concentration of residual COD the factors were taken within the limits of study range, at medium dose of H_2_O_2_ and at lower pH. The Table 5 shows optimum solutions. The maximum concentration of H_2_O_2_ is observed as 29.12 mM in the solution number 5 (*i.e.*) pH: 3.0; dose of FeSO_4_: 14.44; the experiments were conducted in the above predicted condition to validate the efficiency of the model and the results are shown in the Table 6. The predicted concentration of residual COD is 64.86 mg L^−1^ (log_10_ = 1.812) whereas the experimental concentration of residual COD is 64.56 mg L^−1^ (log_10_ = 1.810). The maximum reduction in the organic contaminants is achieved with increase in ferrous ion dose and hydrogen peroxide dose up to some extent, the optimum molar ratio of H_2_O_2_ to Fe^2+^ was found to be in the range of 1.5 to 3.0.^10^

**Table:** Solutions for the degradation of landfill leachate by oxidative Fenton's process – optimised through RSM CCD

Number	pH	Dose of H_2_O_2_	Dose of FeSO_4_	log_10_ (residual COD) transformed scale	Residual COD (mg L^−1^) original scale
1	3.00	30.00	10.00	1.838	68.86
2	3.00	30.00	10.21	1.835	68.39
3	3.00	30.00	10.77	1.827	67.14
4	3.00	29.77	12.52	1.812	64.86
**5**	**3.00**	**29.12**	**14.44**	**1.812**	**64.86**

**Table:** Comparison of predicted response from RSM at original and transformed scale with experimental response

Response	log_10_ (COD) transformed scale	COD (mg L^−1^) original scale
Predicted	1.812	64.86
Experimental confirmation	1.810	64.56

### 3.4 Reduction in TC, TOC, IC

The reduction in Total Organic Carbon (TOC), Inorganic Carbon (IC) and Total Carbon (TC) was observed in the treated sample and is shown in the Table 7. Before the treatment, TOC of the sample was observed as 825 mg L^−1^ and after treatment it was reduced to 212.7 mg L^−1^. Interestingly, complete removal of inorganic carbon was observed after treatment. Before treatment, the ratio between the COD and TOC is 3.6 whereas the ratio decreased to 0.3 after treatment. In accordance with reported literature, TOC removal was less while comparing with the COD removal, it was due to formation of new species of organic acids after oxidation of the organic substance.^27^

**Table:** Reduction in organic, inorganic and total carbon: before and after treatment

Parameter	Before treatment	After treatment	Removal efficiency%
Total organic carbon (TOC) (mg L^−1^)	826	212.7	74.24%
Inorganic carbon (IC) (mg L^−1^)	1059	0.3349	99.96%
Total carbon (TC) (mg L^−1^)	1885	213	88.7%

### 3.5 GC-MS analysis

**Fig. 9:**
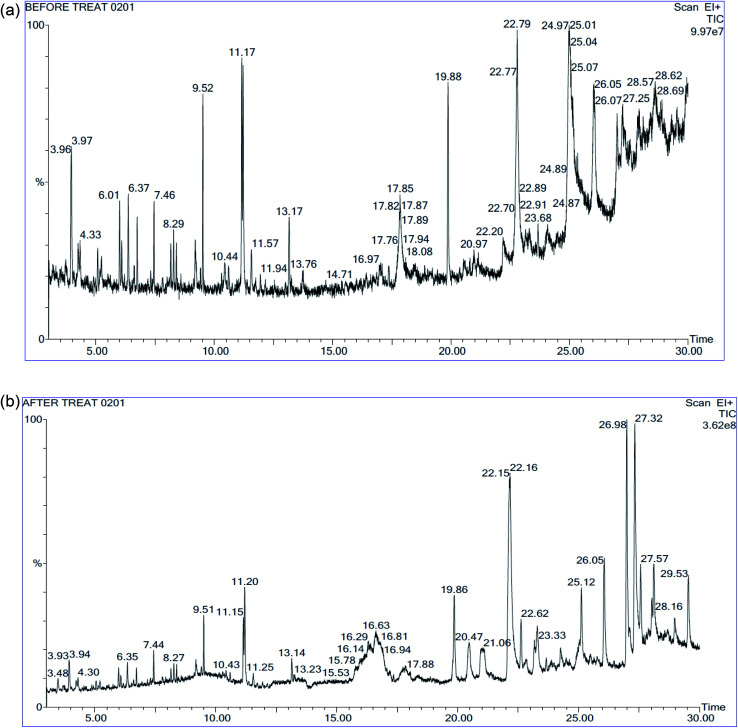
(a) GC-MS spectra of landfill leachate: before treatment. (b) GC-MS spectra of landfill leachate: after treatment.

The GC-MS spectrum result and the peak area of trace organic compounds present in landfill leachate before and after degradation were given in the Fig. 9(a), (b) and Table 8 respectively. Totally 34 different organic substance was identified in the leachate samples before treatment; out of this 24 compounds were not deducted in the samples after treatment as these compounds were completely mineralized by Fenton's process the bond energy of O–H is equal to 109 kcal mol^−1^ which oxidize the lower bond energy compounds such as C–H.^31^ The remaining 10 compounds were partly oxidized as the reduction in the peak area was observed. From the Table 8 most of the compounds of nonlinear ring structure were completely mineralized, it results in the formation of acidic components by breaking the aromatic ring. The formation of acids substantiates such as hexadecanoic acid, ethyl ester was found in the degraded samples decreases the pH; other short chain structures were mineralized partly. Addition to oxidation by hydroxide radicles, COD reduction was due to increase in size of organic substance by shift of dissolved substance to colloidal substance through polymerization and precipitation takes place due to complexation of organic substance with ferric ion.^8^

**Table:** Trace organic compound in landfill leachate^a^

Organic compound	Peak Area%	
	Before	After
3-Carene	1.619	0.596
Cyclohexene, 1-methyl-5-(1-methylethenyl)-	0.523	ND
4-Methylcatechol	0.435	ND
Benzene, 1,3-bis(1-methylethyl)-	0.59	ND
Benzene, 1,4-bis(1-methylethyl)	0.835	0.385
Tridecane	0.434	ND
Benzene, 1,3-bis(1,1-dimethylethyl)	0.584	0.312
l-Glutamine	0.658	0.348
Tetradecane	1.143	0.532
Butylated hydroxytoluene	1.872	0.833
2,4-Di-*tert*-butylphenol	2.08	1.439
Benzoic acid, 4-ethoxy-, ethyl ester	0.555	ND
Hexadecane	0.718	0.547
Dodecaethylene glycol monomethyl ether, TBDMS derivative	0.703	ND
Butanedial, bis[(3-hydroxy-4-methoxyphenyl)methylene]-	0.471	ND
8,14-*Seco*-3,19-epoxyandrostane-8,14-dione,17-acetoxy-3*á*-methoxy-4,4-dimethyl-	0.515	ND
4-Acetyloxyimino-6,6-dimethyl-3-methylsulfanyl-4,5,6,7-tetrahydro-benzo[*c*]thiophene-1-carboxylic acid methyl ester	0.6	ND
14-Methyl-14-(3-oxobutyryloxy)-hexadec-15-enoic acid, methyl ester	0.454	ND
Hexadecanoic acid, ethyl ester	ND	0.497
Heptaethylene glycol	11.566	0.561
Hexaethylene glycol, TBDMS derivative	1.499	5.440
Chromone, 5-hydroxy-6,7,8-trimethoxy-2,3-dimethyl-	1.293	ND
4-[4-(2-Methoxyphenyl)-1*H*-pyrazol-3-yl]benzene-1,3-diol	0.41	ND
Prost-13-en-1-oicacid,9-(methoxyimino)-11,15-bis[(trimethylsilyl)oxy]-,trimethylsilyl ester,	0.754	ND
Cyclononasiloxane, octadecamethyl-	3.876	ND
3,6,9,12-Tetraoxatetradecan-1-ol, 14-[4-(1,1,3,3-tetramethylbutyl)phenoxy]-	0.733	ND
2-[2-[2-[2-[2-[2-[2-[2-[2-[2-(2-Hydroxyethoxy)ethoxy]ethoxy]ethoxy]ethoxy]eth oxy]ethoxy]ethoxy]ethoxy]ethoxy]ethanol	1.417	ND
3,9-Epoxypregnane-11,14,18-triol-20-one, 16- cyano-3-methoxy-, 11-acetate	1.218	ND
*t*-Butyl-(2-[3-(2,2-dimethyl-6-methylenecyclohexyl)-propyl]-[1,3]dithian-2-yl)-dimethyl-silane	0.853	ND
Glycine, *N*-[(3*à*,5*á*)-24-oxo-3-[(trimethylsilyl)oxy]cholan-24-yl]-, methyl ester	0.66	ND
1-Monooleoylglycerol, 2TMS derivative	0.649	ND
(2*S*,2′*S*)-2,2′-Bis[1,4,7,10,13-pentaoxacyclopentadecane]	1.1	ND
4-(*cis*-2,3,4,*trans*-6-Tetramethyl-3-cyclohexenyl)butan-2-one-2,4-dinitrophenylhydrazone	2.164	ND
Corynan-17-ol,18,19-didehydro-10-methoxy-	0.597	ND
Glafenin	0.423	ND

a ND – not deducted.

## 4. Conclusion

Fenton's process is applicable for treating organic substance; the reaction kinetics is faster compared to biological treatment. The destruction of organic substance by degradation is possible due to oxidation rather than transfer of contaminants from one phase to other phase in coagulation or adsorption process. From this study it can be concluded that the Fenton process is suitable for mineralization of organic matter in stabilized leachate. The hydrogen peroxide dose, ferrous sulphate dose, pH was influenced on Fenton process. At optimum condition of Fenton process (pH 3, 29.12 mM H_2_O_2_, 14.44 mM FeSO_4_) COD removal was 97.83% and TOC removal was 74.24%. From GC-MS analysis around 24 number of organic substance was completely mineralized and reduction in peak area (23% to 95%) was observed in remaining 9 organic compounds.

## Conflicts of interest

All the authors hereby declare that, we don't have a conflict of interest.

## Supplementary Material
